# Programmed Cell-Death by Ferroptosis: Antioxidants as Mitigators

**DOI:** 10.3390/ijms20194968

**Published:** 2019-10-08

**Authors:** Naroa Kajarabille, Gladys O. Latunde-Dada

**Affiliations:** 1 Nutrition and Obesity Group, Department of Nutrition and Food Sciences, University of the Basque Country (UPV/EHU), 01006 Vitoria, Spain; naroa.kajarabille@ehu.eus; 2 King’s College London, Department of Nutritional Sciences, Faculty of Life Sciences and Medicine, Franklin-Wilkins Building, 150 Stamford Street, London SE1 9NH, UK

**Keywords:** ferroptosis, antioxidants, glutathione, iron

## Abstract

Iron, the fourth most abundant element in the Earth’s crust, is vital in living organisms because of its diverse ligand-binding and electron-transfer properties. This ability of iron in the redox cycle as a ferrous ion enables it to react with H_2_O_2_, in the Fenton reaction, to produce a hydroxyl radical (•OH)—one of the reactive oxygen species (ROS) that cause deleterious oxidative damage to DNA, proteins, and membrane lipids. Ferroptosis is a non-apoptotic regulated cell death that is dependent on iron and reactive oxygen species (ROS) and is characterized by lipid peroxidation. It is triggered when the endogenous antioxidant status of the cell is compromised, leading to lipid ROS accumulation that is toxic and damaging to the membrane structure. Consequently, oxidative stress and the antioxidant levels of the cells are important modulators of lipid peroxidation that induce this novel form of cell death. Remedies capable of averting iron-dependent lipid peroxidation, therefore, are lipophilic antioxidants, including vitamin E, ferrostatin-1 (Fer-1), liproxstatin-1 (Lip-1) and possibly potent bioactive polyphenols. Moreover, most of the enzymes and proteins that cascade or interact in the pathway of ferroptosis such as a subunit of the cystine/glutamate transporter x_c_^−^ (SLC7A11), glutathione peroxidase 4 (GPX4), and the glutamate–cysteine ligase (GCLC) iron metabolism genes *transferrin receptor 1* (*TfR1*) *ferroportin*, (*Fpn*) *heme oxygenase 1* (*HO-1*) and *ferritin* are regulated by the antioxidant response element of the transcription factor, Nrf2. These, as well as other radical trapping antioxidants (RTAs), are discussed in the current review.

## 1. Introduction

Iron is vital and indispensable for many metabolic and physiological functions in organisms. Life in the primordial age relied on a surfeit of iron, which later became limiting due to the release of oxygen during photosynthesis. Hence, the emergence of complex eukaryotic life on Earth at a relatively low level of oxygen and cold temperature was a challenge that led to the ligation of cysteine to an iron–sulphur scaffold, and unsaturated fatty acids kept the membrane lipid bilayer viscous [1,2]. The highly reactive cysteine thiol groups and fatty acid unsaturation became problematic in the presence of oxygen, and both had to be mitigated against [3]. Subsequently, glutathione emerged to attenuate the reactivity of cysteine thiyl radicals, and reciprocally, cysteine availability from cystine is essential to maintain a high glutathione level (2–10 mM) to protect membrane lipids. Moreover, selenocysteine, the 21st proteinogenic amino acid, also appeared, and its indispensability to the catalytic function of glutathione peroxidase 4 (GPX4) was alluded to recently [3]. The interactions of these players and the orchestration of the processes culminate as a panacea to mitigate fatty acid unsaturation and lipid peroxidation, which ultimately was identified in 2012 as ferroptosis [4,5]. However, prior to this seminal discovery, cysteine deprivation, glutathione depletion and iron-free serum have been associated both in vitro and in vivo with a cell death process that is reversible with lipophilic antioxidants [6,7,8,9], notably including the consequence of the presence of oxygen-facilitated lipid peroxidation which nature counters by chain-breaking measures such as the activity of the selenoenzyme GPX4 and its cofactor, glutathione [10]. It has since been proven that any derangement that occurs to the normal homeostatic regulation of this process leads to ferroptosis, a non-apoptotic regulated cell death that is characterized by iron-induced lipid peroxidation of membranes [5]. Ferroptosis has thus been implicated in various disorders including neurodegenerative diseases, acute renal failure and cancer, amongst others [5]. It has been characterized in this context as a defect in antioxidant defenses by chemical or mutational inhibition or the inactivation of system x_c_^−^ (SLC7A11 and SLC3A2). Thus, the inhibition of GSH synthesis via a decreased uptake of cystine inactivates the GPX4, thereby leading to the accumulation of lipid peroxides. Strategies to ameliorate lipid peroxidation by antioxidants and iron chelators are discussed in the current review.

## 2. Oxidative Stress and Antioxidants

**Figure 1:**
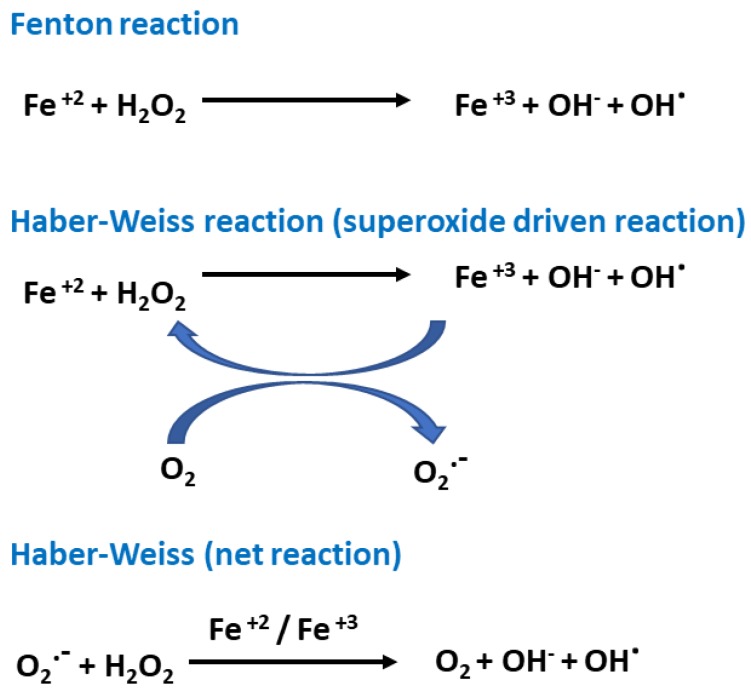
Basic free radical mechanisms of the Fenton and Haber–Weiss reactions.

Oxygen is indispensable to life, but excess oxidants in cellular metabolism are toxic and damaging to tissues and organs. Reactive oxygen species (ROS) are a heterogeneous chemical class that includes hydroxyl radicals (^•^OH) and superoxide anions (O2^•−^), as well as non-radical species such as hydrogen peroxide (H_2_O_2_) [11]. ROS are produced as a result of normal intracellular metabolism in mitochondria and in the endoplasmic reticulum and peroxisomes in the cell, as well as from different cytosolic enzyme systems such as NADPH oxidases (NOX). There are also other enzymatic activities including lipoxygenase (LOX), cyclooxygenase (COX), xanthine oxidase (XO), and nitric oxide synthase (NOS), and cytochrome P450 [12]. Hydroxyl radicals are generated from hydrogen peroxide during cellular oxygen metabolism via the Fenton and Haber–Weiss reactions (Figure 1).

While ROS remain at optimal concentrations and under homeostatic balance, they can play a role as second messengers, contributing to the control of several cellular functions, such as cell survival, growth or differentiation and regulating cellular signaling. However, ROS can cause DNA damage, protein denaturation, and lipid peroxidation when concentrations are above the critical levels. Antioxidants are compounds that are capable of reducing or terminating the destructive chain reactions that are caused by ROS at a minuscule level. Antioxidants such as superoxide dismutase (SOD), catalase (CAT), and glutathione peroxidase (GPX) are the first line of defense against an excess of ROS. When adequate redox homeostasis is compromised, due to decreased antioxidant activity or high amounts of ROS, the cell undergoes cell death [12].

## 3. Rustiness of Membrane Polyunsaturated Fatty Acids (PUFAs)

Lipid peroxidation is a process by which oxidants such as free radicals or non-radical species abstract one of the labile hydrogen atoms from bis-allylic position methylene groups in polyunsaturated fatty acids (PUFAs) and in which oxygenation leads to the accumulation of lipid peroxyl radicals and hydroperoxides [13,14]. Critically, iron is essential to the three different mechanisms of lipid peroxidation pathways, namely (a) a non-enzymatic iron-catalyzed free radical autoxidation chain reaction of lipids, (b) the enzymatic oxidation of lipids primarily mediated by the non-heme iron-containing enzyme lipoxygenase (15-LOX) family [15,16,17], and (c) iron-driven oxidative cleavage or truncation of hydroperoxy-products that could induce nucleophilic damage to proteins capable of causing cell death [18]. While oxidized phosphatidylethanolamines (PE) harboring arachidonoyl (AA) and adrenoyl moieties (AdA) have been shown to be proximate executioners of ferroptosis [13,14,19], the downstream molecular mechanisms of lipid peroxidation and ferroptosis (ultimate proteins or processes that damage membranes) are still conjectural and debatable. Several hypotheses have been proposed, and these include decreased cell membrane thickness and thinning, an accelerated influx of oxidants, destabilized membrane structure and fluidity leading to pore and micelle formation [19,20], promotion of lipid membrane perforation and pore formation [21,22], and the breakdown of lipid hydroperoxides to 4-hydroxy-2-nonenals (4-HNEs) or malondialdehydes (MDAs)—toxic aldehydes capable of crosslinking to undermine membrane structural integrity in ferroptosis [23]. The deleterious consequences of lipid peroxidation in ferroptosis are neutralized and nullified by natural and synthetic antioxidants and iron chelators [5,13].

## 4. Detoxification of Excess Lipid Peroxides and Antioxidant Recycling

### Depletion of Glutathione and Ferroptosis

**Figure 2:**
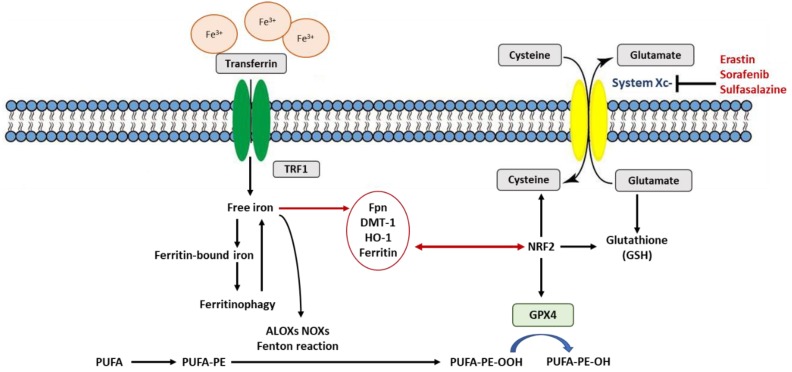
Schematic representation of the molecular pathways of ferroptosis regulation. The indicated pathways regulate ferroptosis sensitivity. Abbreviations: TFR1: transferrin receptor1; Fpn: ferroportin; DMT-1: divalent metal transporter 1; HO-1: Heme oxygenase 1; GPX4: glutathione peroxidase 4; PUFA: polyunsaturated fatty acids; PUFA-PE: polyunsaturated fatty acid containing phosphatidylethanolamine; PUFA-PE-OOH: polyunsaturated-fatty-acid-containing-phospholipid hydroperoxides which cause ferroptosis and is neutralized and reduced by GPX4 to PUFA-PE-OH. ALOXs: arachidonate lipoxygenases; NOXs: Reduced form of nicotinamide adenine dinucleotide phosphate (NADPH) oxidase.

Ferroptosis is defined as an iron-dependent form of regulated cell death, which occurs through the lethal accumulation of lipid-based reactive oxygen species (ROS) when glutathione (GSH)-dependent lipid peroxide repair systems are compromised [4,14]. System x_c_^−^ is a disulfide-linked heterodimer composed of SLC7A11 and SLC3A2 which imports cystine, the extracellular oxidized form of cysteine, in exchange for intracellular glutamate antiporter. GSH is a tripeptide antioxidant that contains the amino acid cysteine, a cofactor for GPX4, the sole selenoenzyme that catabolizes the reduction of phospholipid hydroperoxides [24,25]. Consequently, the inhibition of system x_c_^−^ by erastin decreases the influx of cysteine and causes the intracellular depletion of GSH levels [5]. While the depletion of GSH induces ferroptosis, the enhancement of cysteine synthesis by *trans*-sulfuration is refractory to erastin-induced ferroptosis. Ironically, GSH is presumed to be an evolutionarily ancient protein that renders reactive cysteine thiol groups safe and tolerable to life [2]. In essence, this adaptation neutralizes thiyl radicals and thiol toxicity in the presence of oxygen. The depletion and inhibition of GSH antioxidant levels inactivate and repress the decomposition of lipid peroxides into lipid alcohols to initiate ferroptosis. The induction of ferroptosis is thus induced by the inhibition of cysteine uptake, decreased GSH levels or inactivation of the lipid repair enzyme GPX4. Moreover, GPX4 inhibition due to GSH depletion can, in addition to erastin, be induced by L-buthionine sulfoximine (BSO), sorafenib, sulfasalazine, artesunate, and glutamate, amongst others [5,13]. This is achieved by the inhibition of system x_c_^−^ SLC7A11, the cystine–glutamate antiporter [13], and the inactivation of enzymes such as glutamate–cysteine ligase and glutathione S-transferase that are involved in GSH synthesis and lipid ROS detoxification (Figure 2). However, the direct depletion or inactivation of GPX4 is effected by RAS-selective lethal 3 (RSL3) and wathaferin A, while ferroptosis inducing agent 56 (FIN 56), lovastatin acid and simvastatin inactivate squalene synthase and 3-hydroxy-3-methylglutaryl-coenzymeA (HMG-CoA) reductase, the enzymes involved in lipid hydroperoxide elimination [5,26,27]. Ferroptosis, a process favored by the chemical or mutational inhibition of the cystine–glutamate antiporter, culminates in the accumulation of reactive oxygen species (ROS) in the form of lipid hydroperoxides [28]. Consistent with the above findings, the homozygous knock-out of *Gpx4* is lethal in animals, causing death at the embryonic stage [29], whereas heterozygous animals exhibit high mortality after γ-irradiation [25]. Moreover, the conditional ablation of *Gpx4* in neurons resulted in rapid motor neuron degeneration and paralysis in mice [20]. *Gpx4* inactivation in mice also resulted in acute renal failure [29], which was impeded and attenuated with liproxstatin-1. Furthermore, other ferroptosis inhibitors such as ferrostatin-1 and its more stable and potent analogue (named 16–86) mitigated tissue damage in a model of ischemia/reperfusion injury in both the liver and kidney [13,30]. Ferroptosis has also been implicated in pathologies of the central nervous system, where lipid peroxidation and malfunctioning of GSH metabolism are associated with various neurodegenerative diseases including Parkinson’s disease, Alzheimer disease, Huntington’s disease and Friedrich ataxia [3]. These pathologies and others have been associated with the pivotal role of the nucleophile catalytic moiety of the selenocysteine on the active site of GPX4 [3]. Glutathione is essential for the maintenance of membrane integrity by inhibiting and repairing membrane protein cysteine oxidation and the elimination of electrophiles arising from lipid peroxidation [2]. This function can only be partially redundant in the presence of a hydrophobic, membrane-restricted chain-breaking antioxidant such as α-tocopherol (vitamin E) or phenothiazine [2,31].

## 5. Vitamin E (α-Tocopherol) Is a Potent Peroxyl Radical Scavenger in Ferroptosis

Radical-trapping antioxidants (RTAs) are molecules that break the autoxidation of chain-propagating peroxyl radicals [32]. RTAs protect hydrocarbon biological systems from oxidation and membrane damage. One key example is α-tocopherol, a potent peroxyl radical scavenger that inhibits phospholipid hydroperoxide formation [33,34]. The tocopherols have a high affinity for unpaired electrons and are able to break a cascade of chain reactions in the lipid peroxidation of membranes, and it has been shown that α-tocopherol suppresses ferroptosis in vitro [3]. Vitamin E (α-tocopherol) and α-tocotrienol were shown to regulate ferroptosis via LOX inhibition [35]. They inhibit LOX activity also by competing at the substrate-binding site and by scavenging hydroxyl group radicals. Esterified analogues of vitamin E, α-tocopherol succinate (TS) or α-tocopherol phosphate (TP), though unable to suppress LOX activity, nevertheless can also compete for PUFA substrate-binding sites (the corking mechanism), even without generating tocopheroxyl radicals [17]. Vitamin E has been shown to protect cells against ferroptotic death in vitro [13,36] and in vivo in *Gpx4*−/− knockout mice [17,37]. Quite interestingly, a recent study [38] showed that the ablation of *Gpx4* resulted in perturbed reticulocyte maturation due to uncontrolled lipid peroxidation, and the phenotype is masked by dietary vitamin E supplementation. Hence, vitamin E and GPX4 synergistically hinder lipid hydroperoxides in cells and animal models [39,40]. The tissue-specific expression of 15-LOX and possibly vitamin E status underline disparate organ disorders that are associated with ferroptosis. Tocopherol deficiency results in neurological disease [34], which becomes lethal when selenium is lacking [41] Besides this, deficiency in vitamin E has been linked to the premature development of neurodegeneration, which is associated with ferroptosis [32,42].

Synthetic compounds such as nitroxide (Tempo) are also capable of permeating cell membranes to reduce the oxidative damage caused by ROS. Tempo was shown to inhibit the production of hydroxyl radicals by oxidizing the iron(II)-citrate to iron(III)-citrate, thereby blocking the Fenton reaction in mice [43]. However, the antioxidant mechanism of nitroxides remains as yet undefined [43]. Other LOX inhibitors can also act as antioxidant-trapping radicals to prevent lipid peroxidation [44,45]. These include ebselen and coenzyme Q. Furthermore, the GPX4-generated phenoxyl radical was shown to have a high affinity to react with HL 60 cell homogenate [46] or in intact cells [47]. Interestingly, vitamin E, which is recycled and regenerated by GSH and with vitamin C, can act synergistically to maintain redox homeostasis and prevent lipid peroxidation [48,49]. Hence, ascorbate could act in a network with vitamin E and GSH to confer potent protection by scavenging free radicals by forming ascorbyl radicals, which are often dismutated in a chain-breaking reaction [50]. However, as with iron, ascorbate is a double-edged sword which reacts with molecular oxygen in the presence of ferrous iron (Fe^2+^) to form a superoxide anion (O^2.−^), which in turn forms hydrogen peroxide (H_2_O_2_) [51,52]. Prior to the nomenclature of ferroptosis, dehydroascorbate and 1′-methyl-ascorbic acid, which were oxidized and derived from ascorbate, protected against a programmed ferroptotic-type cell death in the liver [53] and in lymphocytes [54]. Paradoxically, ascorbate could also act synergistically with sorafenib to induce ferroptosis [55]. In essence, the hormetic nature of ascorbate was demonstrated by Lorincz et al. [56] in which ascorbate inhibited both erastin or RLS3-induced ferroptosis in RAS-mutant HT-1080 cell line, while pharmacologically high doses initiated a type of cell death disparate from ferroptosis in the cells [56].

Although vitamin E is a natural RTA, it is comparatively less potent than the two aromatic amines ferrostatin-1 (Fer 1) and liproxstatin-1 (Lip 1) that were identified from a high-throughput screening library. Shah et al. [44] also showed that Fer-1 and Lip-1 are highly effective RTAs in lipid bilayers, suggesting that they alleviate ferroptosis by inhibiting lipid autoxidation [44]. Moreover, liproxstatin-1 attenuated *Gpx4* inactivation and acute renal failure in a *Gpx4* knockout (KO) mouse model [13]. Furthermore, diarylamine RTAs, used to prevent the autoxidation of organic compounds such as oils and grease, have also been shown to be potent inhibitors of ferroptosis [44]. The efficacy of these compounds to suppress ferroptosis was dependent solely on their RTA activity in lipid bilayers. Structural analogues, however, lacking RTA activity are ineffective for ferroptotic inactivation. Thus, diarylamine RTAs present an avenue for the synthesis of compounds beyond Fer-1 and Lip-1 for therapeutic purposes in disease management including cancers, neurodegenerative diseases and acute renal failure amongst others. Melatonin (N-acetyl-5-methoxytryptamine) is an endogenous potent antioxidant which exerts ROS-quenching capabilities and was shown to mitigate against hemin-induced ferroptosis-type cell death in platelets [57]. However, unlike vitamin E, melatonin is a terminal antioxidant because it does not undergo redox cycling [58]. Significantly, most of the antioxidant enzymes and iron metabolism proteins, such as GPX4, GCLC (TfR1), ferroportin (Fpn), heme oxygenase 1 (HO-1), and ferritin, are transcriptionally regulated by nuclear factor erythroid 2-related factor 2 (NRF2).

## 6. NRF2 at the Nexus of Antioxidants Regulating Ferroptosis

Cell death induced by ferroptosis is inextricably linked with a dysregulation in redox homeostasis. Hence, in addition to the role of the antioxidant GSH (described above), NRF2 modulates cellular antioxidant response and mitigates electrophilic and oxidative stress. It is a transcription factor that regulates the basal and responsive cytoprotection to oxidative stress. Basal homeostasis of cellular NRF2 is maintained by ubiquitination in complexation primarily with Kelch-like ECH-associated protein 1 (KEAP 1) and Cul3 E3 ubiquitin ligase. During a heightened state of oxidative stress, KEAP 1 is degraded as it is dissociated from NRF2. This allows NRF2 to translocate into the nucleus to initiate the transcription of antioxidant response element (ARE)-containing genes. Principal proteins and enzymes engaged in the induction and inhibition of ferroptosis are encoded by *Nrf2* target genes. Consequently, the inactivation, inhibition and knock-down of *Nrf2* genes enhance ferroptosis in cells [45]. Glutamate–cysteine ligase (GCLC) and a subunit of the cystine/glutamate transporter x_c_^−^ (SLC7A11), both involved in GSH synthesis and GPX4 (inhibitor lipid ROS peroxidation), are regulated by NRF2 [45,59]. Iron metabolism proteins such as transferrin receptor (TfR1), ferroportin (Fpn), heme oxygenase 1 (HO-1), and ferritin, which are also NRF2-regulated, are involved in iron availability and ferroptosis. Ferroptosis is dependent on exogenous (TfR1, DMT1, Zip14) or endogenous sources of iron via the autophagic/lysosomal degradation of ferritin (ferritinophagy) or through the catabolism of heme by HO-1. Out of the iron metabolism proteins that are involved in ferroptosis, ferritin and HO-1, both of which are transcriptionally regulated by *Nrf2*, exert effects that are equivocal, and research is required in different model systems. Furthermore, *Nrf2* expression could also be enhanced by inducers of ferroptosis to promote the death cell process.

## 7. Is Ferritin an Antioxidant or an Autophagic Ferroptotic Agent?

Ferritin is an iron storage protein that can store up to 4500 Fe(III) ions as ferric-oxide–phosphate in its central core. As nature’s iron storage protein, ferritin, especially H-ferritin, exhibits ferroxidase activity, the product of which, namely ferric ion, is incorporated into the ferritin structure [60] This constitutes a mopping-up exercise that rids cells of excess reactive ferrous ion. The ferroxidase activity of the H-chain sequesters excess cytosolic iron, and this confers antioxidant and cytoprotective functions against oxidative stress [60]. On the other hand, ferritin, as a storage protein, is deployed to deliver the metal into circulation during insufficiency, in particular during the essential survival process of erythropoiesis [61] and when it serves as a source of iron by endocytosis to oligodendrocytes in the brain [62]. Ferritinophagy is the autophagic degradation of the iron-storage ferritin protein that maintains homeostasis during iron depletion. The initiation of ferroptosis was shown to activate ferritinophagy [63] in a process involving ferritin catabolism to increase the labile iron pool (LIP) which promotes ROS accumulation, which drives ferroptosis [28]. The inhibition of nuclear receptor coactivator 4 (NCOA4), an autophagy cargo receptor that binds ferritin heavy chain 1 (FTH1) for lysosomal degradation, repressed ferritin degradation and suppressed ferroptosis, while its overexpression had the opposite effects [20,21]. Previously, it was shown that ferritin at physiological pH is reduced by ascorbate, which was shown to stimulate lipid peroxidation [64,65]. The dual role of ascorbic acid is also evident when it enhances redox cycling in Fenton chemistry (described above) with an increase in the labile iron pool that can promote oxidative damage to DNA, proteins, and lipids as in ferroptosis [66].

## 8. Is Heme Oxygenase (HO-1) an Antioxidant That Protects or Promotes Ferroptosis?

Heme oxygenase catalyzes the enzymatic degradation of heme into biliverdin, ferrous iron, and carbon monoxide. Both HO-1 and biliverdin as Nrf2 target genes antioxidants under specific conditions [67] have been shown to be protective against various stress-related conditions. Moreover, HO-1 has been shown to exert anti-cancer, anti-inflammatory, anti-apoptotic, antiproliferative, and antioxidant properties [26]. It is induced in response to cellular stress, and being cytoprotective, studies have shown that it mitigates ferroptosis in renal proximal tubule cells [68]. In that study, carried out by Adedoyin et al. [68], the knockout of HO-1 promoted erastin-induced ferroptosis in the kidney cells compared to cells overexpressing HO-1. Ferroptosis was also increased by erastin and sorafenib in HO-1 knockout hepatocellular carcinoma cells [69]. The duality of HO-1 as an inducer of ferroptosis derives from its function as an intracellular source of iron, which sequentially initiates ferritin expression to mop up released labile iron. The overexpression of HO-1 accelerates erastin-induced ferroptotic cell death in HT-1080 fibrosarcoma cells [70] and in cancer cells due to the mediation of redox regulation involving endoplasmic reticulum stress and mitochondrial homeostasis [71]. During increased pro-oxidant conditions, iron released from ferritin or heme by HO-1 increases ROS levels, which induce lipid peroxidation and ferroptosis, which could mitigate tumorigenesis. The overactivation of HO-1 may become detrimental and cytotoxic due to increased intracellular iron arising from iron stores, which induces ferroptosis [71], which is detrimental to degenerative diseases. The Janus face of the heme oxygenase/biliverdin reductase system in pathologies is also due to its ability to increase the intracellular levels of pro-oxidant heme, which promotes equimolar amounts of antioxidants and the free radical scavengers biliverdin-IX alpha (BV)/bilirubin-IX alpha (BR), as well as carbon monoxide (CO) and ferrous iron [71]. These levels of cellular iron and ROS shift the equilibrium of HO-1 from a protective role to an executor of cell death [72]. There is, therefore, the prospect of applying HO-1 for the mediation of ferroptosis as a chemotherapeutic strategy against tumors [16,30], even though it is detrimental in leading to disorders such as kidney injury [73] and neurodegeneration.

## 9. Polyphenols as Antioxidants and Iron Chelators 

Polyphenols, as secondary metabolites of plants, are non-nutrient components of human diets, are attracting considerable interest due to their important bioactive ingredients, and are emerging as effective alternatives to synthetic compounds or drugs for the prevention and treatment of a variety of disorders [74] due to their antioxidant propensity. Although polyphenolic compounds possess beneficial therapeutic properties against numerous disorders, the mechanisms of action of most of them are not fully known. In addition to their biological activities, they can serve as scaffolding blocks for the preparation of various functional products such as capsules, antibacterial and antioxidant films, or nanoparticles for compounds in drug delivery [20].

Since ferroptosis is an iron-dependent and reactive oxygen species (ROS)-reliant cell death that is induced by the glutathione-dependent antioxidant defenses, lipophilic antioxidants [75] and iron chelators are potent candidate antidotes. Polyphenols, therefore, could logically serve as agents to avert the lipid peroxidation and cell death caused by ferroptosis.

Curcumin has emerged as a potent antioxidant agent with radical scavenging properties that have been found to have therapeutic benefits in several oxidative stress models [75,76]. For example, [77] demonstrated the therapeutic effect of curcumin in a rhabdomyolysis-induced acute kidney injury (AKI) mouse model of ferroptosis. Curcumin treatment was found to decrease renal dysfunction, lipid peroxidation, inflammation, endothelial damage, and tubular cell death in the kidney of these mice. The antioxidant enzyme heme oxygenase-1 (HO-1) was enhanced by curcumin in this study and may contribute to the mechanism of its therapeutic function. Similarly, curcumin-loaded nanoparticles (Cur-NP) were used to treat rhabdomyolysis and glycerol-induced AKI symptoms in vivo in mice [20]. Cur-NP also countered oxidative stress, growth inhibition and cell apoptosis in vitro in HK-2 cells. Baicalein was demonstrated to exert protection against erastin-induced ferroptosis in exocrine BxPc3 and PANC1 pancreatic cancer cells. The treatment of the cells with baicalein suppressed ferroptosis by inhibiting glutathione depletion, GPX4 degradation and lipid peroxidation. Baicalein may possibly activate the NRF2 pathway and was found to have prevented erastin-induced NRF2 degradation and inhibited oxidative injury in PANC1 cells [78].

Moreover, Kose et al. [79] showed the protective function of (−)-epigallocatechin-3-gallate (EGCG) and curcumin against erastin-induced ferroptosis in mouse insulinoma cells (MIN6) pancreatic β-cells. EGCG and curcumin protected the cells against erastin-induced ferroptosis, similar to baicalein, by preventing iron accumulation, GPX4 inactivation, GSH depletion and lipid peroxidation [79].

Overall, these findings indicate that polyphenolic compounds can prevent lipid peroxidation in cell membranes by acting as ROS scavengers, possibly via the activation of the NRF2 antioxidant proteins. Mechanistically, polyphenols could prevent ferroptosis by chelating or sequestering iron, or may serve as an electron or a hydrogen atom donor in antioxidant reactions. As such, polyphenols, exerting both functions, could prevent radical chain reactions that initiate and terminate the process of ferroptosis. Thus, polyphenols might be potential therapeutic agents against diseases that are associated with ferroptosis.

In summary, natural antioxidants and polyphenols, as well as their synthetic analogues that are capable of inhibiting lipid peroxidation, are important as therapeutic agents for disorders that manifest ferroptosis. The challenge in the future will include research to investigate the potency of the synergy of combined antioxidant vitamins and bioactive polyphenols in preventing ferroptosis in degenerative diseases. Conversely, since some of these compounds have hormetic properties, dose-response experiments will reveal high pharmacological doses of some pro-oxidants that will initiate or induce ROS or ferrotosis in cancer therapy. Addressing the molecular mechanisms of action in both anti and pro-ferrotoptic agents (Table 1) will enhance the knowledge in this field substantially.

## Figures and Tables

**Table 1:** A summary of some inducers and inhibitors of ferroptosis.

Compound	Mechanism	References
**Inducers**		
Erastin	System x_c_^−^ inhibitor	[5,13]
Sulfasalazine	System x_c_^−^ inhibitor	[5,13]
Sorafenib	System x_c_^−^ inhibitor	[5,13,55,69]
BSO	γGCS inhibitor, depletion of GSH	[5,13]
RSL3	GPX4 inhibitor	[5,26,27]
FIN 56	GPX4 inhibitor	[5,26,27]
**Inhibitors**		
Lip-1	Catalytic RTA, prevention of lipid peroxidation	[13,44,75]
Fer-1	Catalytic RTA, prevention of lipid peroxidation	[13,30,44,75]
Vitamin E	Lipophilic antioxidant compensating GPX4 loss	[2,13,35,36,40,42]
Nitroxide-based compounds	Catalytic RTA, prevention of lipid peroxidation	[43]
Curcumin	Preventing iron accumulation, GPX4 inactivation, GSH depletion and lipid peroxidation	[75,77,79]
EGCG	Preventing iron accumulation, GPX4 inactivation, GSH depletion and lipid peroxidation	[63,79]
Baicalein	Prevention of lipid peroxidation Inhibition of glutathione GSH depletion	[78]

BSO: L-buthionine sulfoximine; γGCS: γ-glutamylcysteine synthetase; GPX4: glutathione peroxidase 4; GSH: glutathione RSL3: RAS-selective lethal 3; FIN 56: ferroptosis inducing agent 56; Lip-1: liproxstatin-1; RTA: radical-trapping antioxidant; Fer-1: ferrostatin 1; EGCG: (−)-epigallocatechin-3-gallate.
